# Chapped Hands

**Published:** 1869-01

**Authors:** 


### CHAPPED HANDS

And cracked finger-ends are a great and often a painful annoyance to good housekeepers in winter time, often rendering sewing and darning impossible. The chapping arises from the soap not being perfectly removed by abundant rinsing of the hands after using it; too often putting the hands in water in cold weather is also a cause of chapping, which would be effectually prevented if after each washing a few drops of honey or suet were rubbed thoroughly into the skin of the hands; but these are objectionable; a few drops of glycerine would answer a far better purpose—a vial of it should be in every house; a dime's worth would last half a winter.

If it can be avoided, the hands should not be put into water the whole day in very cold weather, except on rising and retiring. If the hands were well oiled and an old and loose pair of kid gloves were worn during sleep, it would be of advantage, keeping the skin soft and moist.

### CRACKED FINGERS

Are exceedingly troublesome and painful in winter time, mainly occasioned by being too near the fire in parlor or kitchen. A preventive is found in putting on a pair of gloves when the fire needs arranging or replenishing. To cure the cracking, the expedient and most efficient method is to keep the whole hands well oiled and to wear gloves all the time, keeping the hands away from the fire or heated surfaces and avoiding everything which causes pain to the part affected. The great point as to chapped hands and cracked fingers in cold weather is

1. To keep them as dry as possible.
2. Keep away from the fire.
3. Oil them and sleep in gloves at night.

